# The Role of the Greater Omentum Flap in the Prevention of Low Anterior Resection Syndrome (LARS): A Systematic Review of the Literature

**DOI:** 10.3390/jcm14227937

**Published:** 2025-11-09

**Authors:** Konstantinos Perivoliotis, Ioannis Baloyiannis, Chamaidi Sarakatsianou, George Tzovaras

**Affiliations:** 1 Department of Colorectal Surgery, The Royal Marsden NHS Foundation Trust, London SW3 6JJ, UK; 2 Department of Surgery, University Hospital of Larissa, 413 34 Larissa, Greece; balioan@hotmail.com (I.B.); geotzovaras@gmail.com (G.T.); 3 Accident and Emergency Department, General Hospital of Larissa, 412 21 Larissa, Greece; heidisarak@gmail.com

**Keywords:** omental, flap, low, anterior, syndrome, meta-analysis

## Abstract

**Background/Objectives**: We aimed to evaluate the evidence regarding the effect of greater omentum flap transposition (OFT) on low anterior resection syndrome (LARS) in patients who undergo rectal resections. **Methods:** This study was conducted according to the Cochrane Handbook and the Preferred Reporting Items for Systematic Reviews and Meta-Analyses (PRISMA) guidelines. Quality assessment was based on the ROBINS-I tool. Analyses were performed by utilizing the Random Effects (RE) model. **Results**: Overall, three retrospective studies and 100 patients were included in this meta-analysis. Pooled analysis confirmed that OFT did not improve postoperative LARS scores in 3 months (MD: −1.54; 95%CI: −7.30, 4.22; *p* = 0.6), 6 (MD: 3.75; 95%CI: −20.16, 27.66; *p* = 0.76) and 9 (MD: −4.86; 95%CI: −28.38, 18.66; *p* = 0.69) months postoperatively. Similarly, OFT did not impact the overall postoperative morbidity rate (*p* = 0.1), the pooled operation duration (*p* = 0.21), and the hospitalization duration (*p* = 0.21). **Conclusions**: Our preliminary results suggest that OFT does not influence postoperative LARS, morbidity, and perioperative efficiency. Given the limited available evidence and several study limitations, further randomized controlled trials are required.

## 1. Introduction

Surgical resection is the pivotal treatment modality for several malignant and pre-malignant pathologies of the rectum [1,2]. Recent refinements in the operative approach, the introduction of minimal invasive techniques, and developments in anesthetic protocols have allowed the enhancement of perioperative care and the improvement of long-term survival outcomes [3,4,5].

Over recent decades, colorectal research efforts have also focused on the optimization of postoperative functional recovery after rectal resection [6,7]. This is because in most cases, patients who undergo rectal surgery will develop sexual, urinary, or bowel functional deficit. Regarding the latter, symptoms like stool fragmentation, defecation urgency, and incontinence are frequently reported [6,7]. These comprise part of the well-known Low Anterior Resection Syndrome (LARS), whose severe impact on the patients’ quality of life has been extensively documented [6,7]. The administration of neoadjuvant chemoradiotherapy, the utilization of a defunctioning stoma, and the extent of the surgical resection are among the most prominent risk factors for the development of LARS [6,7].

Total mesorectal excision (TME) contributes to LARS through multiple pathways [6,8,9]. These include intraoperative injury to the autonomic nerve plexuses, interruption of the anal sphincter function, and a reduction in rectal compliance due to postoperative formation of adhesions with the pelvic parietal wall [6,8,9].

To counteract these, filling the pelvis with a well-vascularized omental flap has been described as a valid option [10,11,12]. The omentum is an abundant intraabdominal layer of fat with multiple biological properties, including healing and regulation of local inflammatory response [10,11,12]. Theoretically, the omentum also allows the mechanical reconstruction of the pelvis since it acts as a buffer compliance zone for the neorectum, thus affecting postoperative functional recovery [10,11,12]. Although recent cohorts have attempted to provide clinical evidence regarding the omental flap, there is still no pooled data regarding its efficacy in influencing postoperative LARS [10,11,12].

The aim of this study is to assess the currently available evidence regarding the effect of greater omentum flap transposition (OFT) on postoperative LARS, perioperative safety, and efficacy in patients who undergo rectal resections.

## 2. Materials and Methods

### 2.1. Study Protocol

This systematic review and meta-analysis were performed according to the Cochrane Handbook and the Preferred Reporting Items for Systematic Reviews and Meta-Analyses (PRISMA) guidelines [13,14].

An a priori protocol registration was performed (10.17605/OSF.IO/XKYRJ).

### 2.2. Search Strategy

To identify eligible articles, a systematic screening strategy was applied in major scholar databases (Medline, Scopus, Web of Science, CENTRAL, medRxiv). The last search date was 5 August 2025. The following keywords were used combined with Boolean logic nexuses:

“omental”, “omentum”, “LARS”, “low anterior”, “rectum”, “rectal”

The reference list of the included articles was also reviewed.

### 2.3. Endpoints

The primary endpoint of this study was the comparison of OFT and control in terms of postoperative LARS. The evaluated time endpoints were 3, 6, and 9 months postoperatively.

Secondary endpoints included perioperative safety (overall complications, postoperative ileus, urinary retention, sepsis, anastomotic leakage) and efficacy points (operation duration, length of hospital stay) of interest.

### 2.4. Eligibility and Exclusion Criteria

All human studies reporting on adult patients comparing OFT with control during rectal resections and providing data on the primary outcome of interest were considered eligible.

The following exclusion criteria were considered: (1) non-human studies, (2) studies not reporting data on outcomes of interest, (3) pediatric population, (4) articles in the form of editorials, letters, or conference abstracts, (5) studies with no comparison arm.

### 2.5. Quality Assessment

All eligible studies underwent quality assessment based on the Risk of Bias in Non-Randomized Studies of Interventions (ROBINS-I) tool [15]. This tool grades the methodological quality of studies in the following domains: handling of confounders, patient selection, classification of interventions, deviation from intended interventions, management of missing data, bias in outcome measurement, and selection of reported results. Quality assessment was performed blindly and in duplicate by two independent researchers. Interrater agreement was quantified through Cohen’s K statistic.

### 2.6. Study Selection and Data Collection

Following electronic database screening, duplicate records were removed. The next step included the assessment of compliance of titles and abstracts with the inclusion and exclusion criteria. Following this, a full text review of the remaining articles was performed. Literature screening and data extraction were performed in parallel and blindly by two researchers (K.P., C.S.). In the case of a discrepancy that was not resolved through mutual discussion, the opinion of a third researcher (I.B.) was considered.

Besides the previously mentioned study endpoints, data regarding the study (name of first author, publication date, type of study, country, number of involved centers, study period, number of patients, gender allocation, body mass index—BMI, age, follow up), patient and tumor characteristics (history of previous abdominal operations, inclusion and exclusion criteria, American Society of Anesthesiologists—ASA, tumor node metastasis (TNM) status, perioperative treatment, tumor height), and operative technical details (stoma closure timing, number of surgeons, previous experience, surgical technique, omental flap technique, defunctioning stoma, laparoscopic cases) were extracted.

### 2.7. Statistical Analysis

All statistical analyses were performed in IBM SPSS version 29 (IBM Corp., Armonk, NY, USA) and Review Manager 5.4.1 (The Cochrane Collaboration). Continuous and categorical data were reported as mean (standard deviation—SD) and N, respectively. In cases when these were not available, they were estimated by the respective data (median, range, interquartile range—IQR), using the algorithm proposed by Hozo et al. [16]. Moreover, combined group means and SDs were also calculated [13]. Similarly, if the outcome data were not tabulated, they were extracted from the respective graphs utilizing an electronic extraction software (Digitizelt version 2.6) and a standardized methodology [17].

Pooled continuous outcomes were reported as mean difference (MD), with the corresponding 95% confidence interval (95% CI). The effect size of binary outcomes was the odds ratio (OR), with the respective 95% CI. For the identification of publication bias, the respective funnel plot of the primary endpoint was provided.

Statistical analysis was based on the Inverse Variance (IV) and Mantel–Haenszel (MH) method, respectively. Heterogeneity was estimated through the calculation of I^2^, while Cochran Q test results confirmed the respective significance. The random effects (RE) and fixed-effect (FE) models were both estimated. Due to the small number of eligible studies and the expected high clinical heterogeneity, only the RE model was reported [13]. Statistical significance was considered at the level of *p* < 0.05.

## 3. Results

### 3.1. Search Results

**Figure 1:**
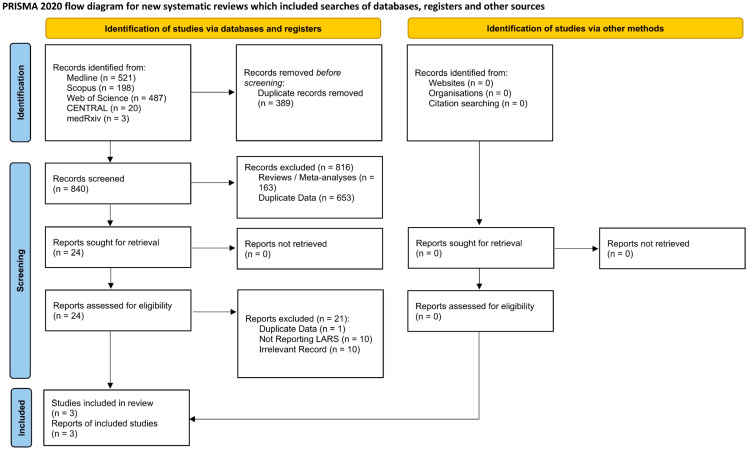
PRISMA flow diagram.

The initial application of the screening algorithm resulted in the retrieval of 1229 records (Figure 1). Following the removal of 389 duplicate entries, 840 studies were submitted for title and abstract review. During this step, 816 articles were rejected. Consequently, 24 manuscripts underwent full text review, with 21 of them (duplicate data: 1 [18]; not reporting on LARS: 10; irrelevant records: 10) being excluded. This led to the identification of three articles [10,11,12] derived from the same research team without fully overlapping study periods. In addition to these, differences in patient characteristics, underlying pathology and inclusion and exclusion criteria were also identified. Therefore, all three studies [10,11,12] were considered eligible and were submitted for qualitative and quantitative synthesis.

### 3.2. Study Characteristics

Table 1 summarizes the characteristics of the included studies. All studies were single retrospective cohorts in nature and performed in China. Publication dates ranged from 2021 to 2023. Overall, 100 patients were included in this meta-analysis. Gender, BMI, and age allocation data are also available in Table 1. The mean postoperative follow-up spanned from 3 to 12 months.

The inclusion and exclusion criteria of each eligible study are provided in Supplementary Materials Table S1. More specifically, regarding the former, the study of Meng et al. [10] considered all patients with low rectal cancers (<5 cm from the dentate line, T1–T3). Qin et al. [12] included all familial adenomatous polyposis cases where a resection was performed. Finally, Liao et al. [11] considered as eligible all stage I–III rectal tumors that were below the peritoneal reflection and within 5 cm above the dentate line and were resected on the principles of TME. Postoperative anorectal follow-up and the absence of a fecal incontinence history were also included.

Similarly, ASA status and TNM classification details are also reported in Supplementary Material Table S1. Overall, 31 cases were introduced in a neoadjuvant treatment protocol, while adjuvant chemotherapy was administered in 40 cases. The mean tumor height ranged from 1.9 cm to 4.77 cm.

All cases were performed utilizing a laparoscopic approach. A defunctioning stoma was provided in 87 cases. Stoma reversal was performed at an average of 17.49 to 18.9 weeks postoperatively (Supplementary Material Table S2). Rectal resection was performed according to the TME principles in all cases. In the study by Qin et al. [12], greater omentum flap transposition was performed in patients who underwent total proctocolectomy and straight ileoanal anastomosis. In the control arm, total proctocolectomy and ileal pouch–anal anastomosis and total colectomy and ileal pouch–rectal anastomosis were performed. The greater omentum flap transposition technique was consistent across studies; it encompassed the detachment from left to right along the gastroepiploic arch and from the transverse colon and the delivery along the parietal wall to fill the presacral space.

### 3.3. Quality Assessment

Quality assessment (Supplementary Material Figure S1) highlighted that all three studies had serious methodological flaws in terms of managing confounders. The interrater agreement was acceptable (Cohen’s K: 81.1%, *p* < 0.001).

### 3.4. Primary Outcome

**Figure 2:**
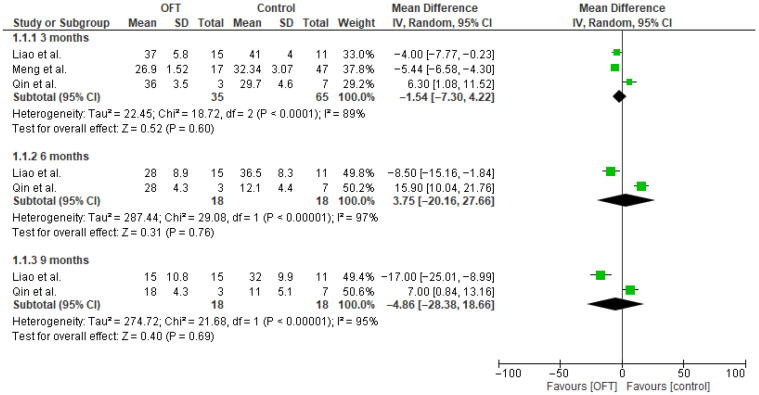
Postoperative LARS score forest plot [10,11,12].

Pooled analysis of the available data confirmed that, compared to the control, OFT did not improve postoperative LARS scores (Figure 2) in 3 months (MD: −1.54; 95%CI: −7.30, 4.22; *p* = 0.6). Similarly, a non-significant effect size was estimated in both 6 (MD: 3.75; 95%CI: −20.16, 27.66; *p* = 0.76) and 9 (MD: −4.86; 95%CI: −28.38, 18.66; *p* = 0.69) months postoperatively. Increased levels of heterogeneity were identified at all time endpoints (I2 at 3 months: 89%; 6 months: 97%; 9 months: 95%).

### 3.5. Secondary Outcomes

OFT did not impact the overall postoperative morbidity rate (Supplementary Material Figure S2; OR: 0.35; 95%CI: 0.1, 1.2; *p* = 0.1). More specifically, the two study groups were comparable in terms of specific complications such as postoperative ileus (OR: 0.37; 95%CI: 0.06, 2.16; *p* = 0.27), urinary retention (OR: 0.35; 95%CI: 0.04, 3.35; *p* = 0.36), sepsis (OR: 1.07; 95%CI: 0.14, 7.94; *p* = 0.95), and anastomotic leakage (OR: 2.47; 95%CI: 0.19, 32.68; *p* = 0.49).

The addition of OFT did not result in an increase in the pooled operation duration (Supplementary Material Figure S3; MD: −16.07; 95%CI: −48.68, 16.55; *p* = 0.21) and did not affect the overall hospitalization duration (MD: −3.42; 95%CI: −8.78, 1.93; *p* = 0.21).

### 3.6. Publication Bias

Visual inspection of the primary outcome funnel plot (Supplementary Material Figure S4) revealed a symmetrical distribution of the eligible studies over the mean effect size, thus suggesting the absence of publication bias. Due to the small number of eligible studies, an Egger’s test was not performed.

## 4. Discussion

### 4.1. Evidence Appraisal

In the early 1980s, Professor Heald standardized and aided the widespread adoption of TME [5]. In the initial description, total mesorectal excision entails the removal of the rectum and its surrounding adipose tissue with all lympho-vascular structures in an intact package through sharp dissection alongside the embryological planes [5]. The application of these principles resulted in a reduction in local recurrence rates and the enhancement of survival rates in patients with rectal malignancies [19].

Dissection under direct vision in the embryologically determined planes of the pelvis allows the mobilization of the rectum from the parietal pelvic structures and the identification and preservation of the autonomic nerves that control bowel, sexual and urinary function [5]. However, even in the case of meticulous dissection, pelvic floor nerve branches will inevitably be affected, causing postoperative functional deficits [10].

It is estimated that almost 60–80% of patients who undergo rectal resection will, ultimately, develop some form of LARS [20]. The reported symptoms of this syndrome often include unpredictable bowel function, altered stool consistency, increased defecation frequency, emptying difficulties, urgency, and some form of incontinence [7]. Consequently, patients become toilet-dependent with a detrimental impact on their mental and emotional welfare and their social life [7].

Several factors including neoadjuvant treatment, tumor stage, defunctioning stoma, and tumor height have been confirmed as key parameters contributing to LARS [6]. Besides these, during TME, disruption to the middle and inferior rectal plexus reduces the postoperative anal resting pressure by intervening in anal sphincter function [10]. In addition to these, the en bloc removal of the rectum and the mesangial tissue results in the formation of fibrous tissue over the newly formed rectum that replaces the normal mesorectal fat [12]. In time, due to the traction between fibrous adhesions and the pelvic sidewall, it leads to inadequate dilatation and compliance in the neorectum [12].

The recovery of rectal compliance is directly correlated with the extent of postoperative LARS [8,9]. After rectal resection and restoration of bowel continuity, rectal distensibility is significantly reduced, and pressure sensitivity is severely impacted [8,9]. In any case, the neorectum will, eventually, undergo gradual expansion over a one-year period; however, this compensation will never reach the preoperative functional levels [8,9,10].

To tackle this issue, the use of OFT during rectal cancer surgery has been described [10,11,12]. The omentum is a fat storage tissue layer that also has regenerative and healing functions [10,11,12]. The transposition of a well-vascularized omental flap prevents inflammation and promotes nerve function recovery [10]. Despite these biological properties, OFT also results in the anatomical reconstruction of the pelvis [10,11,12]. More specifically the use of omentum as a component of the mesorectal fascia inhibits the formation of scar tissue, thus reducing neorectum fibrous attachments to the parietal wall [10,11,12]. Moreover, theoretically, filling the pelvis with omental fatty tissue would act as a cushion, thus enhancing the intestinal buffer force and improving rectal compliance [10,11,12].

An initial study by Meng et al. [10] confirmed that postoperative mesorectal fascia thickness and rectal compliance were negatively associated with the severity of LARS. In addition to these, it was reported that patients receiving OFT had significantly lower LARS scores and improved compliance compared to the control group [10]. In terms of anorectal manometry effects, Liao et al. [11] evaluated OFT in patients with ultra-low rectal cancer and showed that only rectal resting pressure was affected; however, LARS scores retained a significant reduction during the whole follow-up period of 12 months. In the study by Qin et al. [12], though, the addition of OFT in patients who underwent total proctocolectomy and straight ileoanal anastomosis performed worse compared to ileal pouch–anal anastomosis and ileal pouch–rectal cases in terms of defecation frequency and LARS scores. Our pooled data could not confirm the presence of a significant benefit of OFT in LARS scores at any of the three predetermined time endpoints. Furthermore, manometric data were scarce, and so further analyses could not be performed.

As a source of repair biomaterials, the omentum has been utilized in multiple settings as a method of risk mitigation and treatment of postoperative complications [21,22,23]. This has also been the case in colorectal surgery, where the role of OFT has been extensively examined; however, the results have not been encouraging [24,25,26,27]. A French randomized controlled trial with 712 patients concluded that omentoplasty in colonic and rectal resections did not impact the rate of intra or extra-abdominal complications [25]. Similarly, in a recent study by Ozben et al. [24], an omental pedicle flap did not provide any benefit in reducing the risk of postoperative morbidity after rectal cancer surgery. On par with these, our review did not identify a significant benefit of OFT in lessening the overall morbidity burden.

Anastomotic leakage is among the most dreaded postoperative complications. The recent literature suggests that the risk of anastomotic leakage in colorectal surgery ranges from 2% to 19%, with a decrease to 2–7% when specialized surgical teams are involved [28,29]. Multiple perioperative risk factors have been identified including patient characteristics, preoperative treatment, surgical expertise, and tumor distance from the anal verge [28,29]. Regarding the latter, it is estimated that low rectal anastomoses are associated with an almost 24% leakage risk [29]. The development of an anastomotic leakage severely impacts the short-term postoperative recovery, resulting in an increased risk of mortality, reoperation, and the need for a permanent stoma [30,31]; to add to these, long-term sequalae such as chemotherapy delay, risk of recurrence, and decreased overall survival are also present [30,31]. The protective effect of the omentum in reducing anastomotic leakage has been repeatedly investigated. In a study by Agnifi et al. [27], omental wrap during colorectal resections decreased the risk of leakage, reoperation, and mortality. On the contrary a systematic review by Wiggins et al. could not confirm these findings [32]. Similarly, our analyses suggested that OFT did not reduce the risk of anastomotic leaks.

In some initial reports, the use of pedicled omental flaps was associated with an increased risk of postoperative ileus [33,34]. Van Garderen et al. [33] reported the need for operative management of ileus in 3 out of 74 cases where pedicled omental flaps were used. In a comparative study by Klaver et al. [34], in patients on whom a concomitant omentoplasty was performed, a significantly prolonged time was required to remove the nasogastric tube, initiate normal diet, and restore normal bowel function. The theoretical mechanisms involved include a mechanical obstruction or the devascularization of the greater curvature of the stomach that in turn may lead to the alteration of the microcirculation of the Cajal cells [34]. Despite these, we could not validate an increased risk of postoperative ileus in the OFT group.

To perform OFT during rectal resections, the detachment of the greater omentum along the gastroepiploic arch and transverse colon is required [10,11,12]. The next step is the delivery of the flap along the parietal wall and the filling of the presacral space [10,11,12]. During this process, there is a risk of the devascularization of the omentum, leading to flap necrosis. In the cohort of van Garderen et al. [33], flap necrosis was reported in two cases. In our review, none of the eligible studies reported a flap-related complication.

The addition of the OFT surgical steps may result in a significant increase in the duration of surgery in an already extensive operation. In a systematic review by Kileen et al. [26], the performance of various omentoplasty techniques during proctocolectomy led to the addition of an approximate 20 min of operative time. An operation duration difference was not reported in either of the two included studies [11,12]. Subsequently our pooled results could not identify a significant impact of OFT on operative time.

The length of hospital stay is a direct derivative of the extent of an operation and the course of postoperative recovery. Among others, a radical resection or the development of complications may prolong postoperative hospitalization [35]. We estimated that the addition of OFT in rectal cancer surgery did not impact the duration of hospital stay. This was also accompanied by significant levels of heterogeneity; more specifically, in the study by Liao et al. [11], there was no difference between the two groups, while in the report by Qin et al. [12], a mean reduction of 6.5 days was estimated in the experimental arm. It must be noted though that, regarding the latter, different surgical techniques were performed in the two groups, thus limiting the credibility of this result.

Besides recent advancements in surgical techniques and considerable breakthroughs in the diagnosis and classification of LARS, future research should focus on multiple domains of prediction and management [36,37,38]. For instance, artificial intelligence-based prediction models, structured care pathways, neuroplasticity interventions, and novel biomarkers are among the most promising [36,37,38]. The role of ischemia and inflammatory biomarkers in diagnosing and predicting complications and anastomotic leakages has been extensively studied [36]. Additionally, new agents are continuously emerging, and their association with postoperative clinical outcomes is being confirmed [39]. However, this is not the case for LARS, where anatomical and treatment characteristics were mainly used in predictive models [36,37,38]. Acknowledging this literature gap, the direct and indirect prognostic and diagnostic relationship between pharmacodynamic and pathophysiology biomarkers and LARS should be extensively investigated [36,37,38].

### 4.2. Strengths

To the best of our knowledge, this study is the first attempt to summarize the available evidence regarding the effect of OFT on postoperative LARS in patients who undergo rectal resection. It utilized a validated systematic review protocol and applied a methodology that adhered to the respective guidelines. In addition to these, it identified gaps in the relative evidence, thus guiding further research efforts.

### 4.3. Limitations

Several study limitations should be highlighted before the appraisal of our findings. First, our review included only three studies with a small number of patients. This underpowers the statistical analyses and may impede the identification of minimal differences. Moreover, it prohibited the evaluation of further relevant endpoints and the performance of explanatory analyses. In addition, all publications were from the same research team and with partially overlapping study periods. Although the studies evaluated heterogeneous patient populations and introduced different inclusion and exclusion criteria, this may increase the risk of duplicate data. Furthermore, this restrains the ability to extrapolate our data to a broader patient population and surgeons. The base heterogeneity in terms of patient and underlying pathology, including disease type, stage, and tumor height, may also severely impact our results. In addition to these, the operation performed varied significantly between the studies, thus further contributing to the overall bias. Finally, although LARS was assessed using a tool validated in multiple clinical parameters, the fact that it is a patient-reported questionnaire may decrease the significance of our results. This could be addressed by the assessment of the OFT impact on LARS with other tools and anorectal physiology measurements.

## 5. Conclusions

Our study summarized current evidence regarding the effect of OFT on postoperative LARS. Preliminary results suggest that the performance of OFT during rectal resections does not impact postoperative LARS scores. Similarly postoperative morbidity and efficiency outcomes do not improve with OFT. However, due to the small number of eligible studies and severe study limitations, these findings are not definitive and reflect the limited experience in the current literature. Therefore, further randomized controlled trials with an improved methodological profile and a larger sample size are required to delineate the exact effect of OFT.

## Figures and Tables

**Table 1:** Main characteristics of the included studies.

First Author	Publication Date	Type of Study	Country	Single/Multi-Center	Study Period	Group	Number	Male	BMI	Age	Follow-Up (Months)
Meng et al. [10]	2021	Retrospective	China	single	2018–2020	OFT	17	10	20.58 (3.7)	49.82 (14.74)	3
						Control	47	31	21.9 (2.9)	60.6 (9.2)	
Liao et al. [11]	2023	Retrospective	China	single	2015–2022	OFT	15	4	21.23 (3.3)	59 (9.25)	12
						Control	11	5	24.02 (3.7)	57 (10.25)	
Qin et al. [12]	2022	Retrospective	China	single	2015–2021	OFT	3	5	20.83 (2.7)	33 (11.5)	9
						Control	7				

## Data Availability

Data available on request from the authors.

## Supplementary Materials

The following supporting information can be downloaded at: https://www.mdpi.com/article/10.3390/jcm14227937/s1, Table S1. Patient and Tumor Characteristics, Table S2. Operative Technique Characteristics, Table S3. PRISMA Checklist, Figure S1. ROBINS-I Assessment Summary, Figure S2. Postoperative Complications Forest Plot, Figure S3. Perioperative Efficacy Endpoints Forest Plot, Figure S4. Primary Endpoint Funnel Plot.

## Abbreviations

The following abbreviations are used in this manuscript:

PRISMA	Preferred Reporting Items For Systematic Reviews And Meta-Analyses
ROBINS-I	Risk of Bias in Non-Randomized Studies of Interventions
SD	Standard Deviation
IQR	Interquartile Range
95% CI	95% Confidence Interval
MD	Mean Difference
OR	Odds Ratio
IV	Inverse Variance
MH	Mantel–Haenszel
RE	Random Effects
FE	Fixed-Effect
n/a	Not Applicable
BMI	Body Mass Index
ASA	American Society of Anesthesiologists
TNM	Tumor Node Metastasis
TME	Total Mesorectal Excision
FAP	Familial Adenomatous Polyposis
OFT	Omental Flap Transposition
LARS	Low Anterior Resection Syndrome
